# MRI paving the way to diagnosis of acute lymphoblastic leukemia in children and adolescents – a retrospective observational study

**DOI:** 10.1186/s40348-026-00253-0

**Published:** 2026-08-04

**Authors:** Jan Lipp, Jörg Schaper, Franziska Rummel, Melda Kural, Arndt Borkhardt, Florian Babor, Triantafyllia Brozou

**Affiliations:** 1 Department of Pediatric Oncology, Hematology and Clinical Immunology, Medical Faculty, Heinrich-Heine-University Duesseldorf, Moorenstraße 5, Duesseldorf, 40225 Germany; 2 Department of Diagnostic and Interventional Radiology, Medical Faculty, Heinrich-Heine-University Duesseldorf, Moorenstraße 5, Duesseldorf, 40225 Germany

**Keywords:** Childhood leukemia, Musculoskeletal symptoms, MR imaging, Bone marrow signal alterations

## Abstract

**Background:**

Prompt diagnosis of childhood acute lymphoblastic leukemia (ALL) remains a clinical challenge, particularly in children presenting with normal blood counts and musculoskeletal symptoms. With the increasing use of magnetic resonance imaging (MRI) in evaluating such complaints, bone marrow signal alterations may be detected before hematologic abnormalities appear. Previous studies have described characteristic MRI patterns in leukemia, suggesting potential for earlier recognition. This study aims to clarify the role of MRI in the diagnostic pathway of pediatric ALL and to define radiological findings that should prompt bone marrow examination. We retrospectively reviewed all patients diagnosed with ALL at our institution between 2006 and 2024 and included those who underwent MRI prior to diagnosis. Clinical data were collected, and all MRI studies were re-evaluated by an experienced pediatric radiologist.

**Results:**

Twenty pediatric patients with ALL were included. Due to differing MRI protocols, the cohort was divided into two groups: one group (*n* = 17) underwent musculoskeletal imaging and one group (*n* = 3) underwent imaging of the trunk. Overall, MRI-detected bone marrow signal alterations contributed to diagnosis in 20 of 424 (4.7%) newly diagnosed pediatric ALL cases. More than one-third of patients (7/20; 35%) had a completely normal complete blood count (CBC) at presentation. Persistent bone pain was the leading symptom, preceding imaging in 18/20 cases. The mean time from symptom onset to MRI was 20.8 days (range 1–65), while the mean time from MRI to diagnosis was 18.4 days (range 0–180). In the musculoskeletal scans, all patients showed bone marrow signal alterations, predominantly T1-weighted (T1w) hypointensity (17/17) and proton-density-weighted (PDw) or T2-weighted short tau inversion recovery (T2w-STIR) hyperintensity (16/17). In the trunk scans, diffusion-weighted imaging revealed high DWI signal with low ADC values (3/3), and T2-weighted images demonstrated focal lesions (3/3).

**Conclusion:**

MRI plays an important role in the diagnostic pathway of pediatric ALL, especially in patients with persistent musculoskeletal pain and normal blood counts. In our cohort, MRI examinations could initiate a rapid diagnostic process leading to ALL confirmation. Diffuse T1w hypointensity and diffusion restriction emerged as key imaging findings. A normal CBC should therefore not delay urgent oncologic assessment when the clinical presentation and MRI pattern are suspicious for leukemia.

## Background

ALL is the most prevalent malignancy in children, accounting for approximately 25–30% of all pediatric cancers [1]. Leukemia suspicion and diagnosis are based on quantitative as well as morphological abnormalities of peripheral blood and bone marrow. Consequently, the diagnostic process may be complicated in a subset of children, as an entirely normal CBC has been described in approximately 3–10% of childhood ALL cases, depending on the criteria used to define “normal” [2, 3]. A near normal or subtly abnormal CBC (e.g., mild anemia alone, low-normal platelets, no circulating blasts) is even more common, particularly in children presenting with bone pain or musculoskeletal symptoms [4]. Additionally, a large retrospective study including 1,003 patients reported that 14.7% lacked peripheral blood blasts at initial presentation, further complicating the diagnostic pathway [5]. In this context, particularly in children with a normal CBC, musculoskeletal symptoms become more prominent: limb pain is a common presenting feature, occurring in approximately 43% of cases [6]. However, persistent bone pain represents a significant diagnostic challenge, as it may mimic other conditions such as osteomyelitis or juvenile idiopathic arthritis (JIA) [7].

In the context of perplexing clinical overlap with other inflammatory conditions, prior studies have sought clinical, laboratory, and radiologic characteristics that enable earlier identification of children with leukemia [8]. In cases of persistent bone pain for more than one month, cytopenias, especially thrombocytopenia and granulocytopenia, together with an elevated C-reactive protein (CRP) correlate with the presence of ALL and should prompt bone marrow evaluation [9]. Furthermore, novel biomarkers have been explored for their ability to discriminate between ALL and juvenile idiopathic arthritis (JIA). In this context, Brix et al. demonstrated in a cohort of 286 children that interleukins (IL-) 4 and 13 can distinguish between ALL and JIA [10].

In light of the previously described diagnostic dilemma, the use of MRI has increased substantially over recent decades, reflecting its growing importance in the diagnostic workup of clinically ambiguous cases. Indeed, the number of MRI scans performed in Europe more than doubled between 2011 and 2021. Recent statistics from Germany indicate that MRI utilization rose to as many as 158 examinations per 1,000 inhabitants in 2021 [11]. Given that MRI does not involve ionizing radiation, in contrast to computed tomography (CT), it has become a standard imaging modality in pediatric practice. In children with musculoskeletal (MSK) symptoms and absence of peripheral blood count abnormalities, MRI is often used for further investigation. Therefore, MRI is frequently the first diagnostic tool that may lead to suspicion of an underlying leukemia. In adult populations, typical bone marrow alterations in hematological malignancies are well described. Leukemic infiltration may lead to T1w hypointensity and T2w-STIR hyperintensity [12, 13]. In children with leukemia and lymphoma, there is an additional association with aggressive periosteal reactions [14]. Notably, these imaging alterations can precede abnormalities in peripheral blood counts. This is supported by small case series in which children with bone pain and initially normal blood counts underwent bone marrow evaluation after MRI revealed diffuse T1w marrow signal loss [15].

Beyond conventional sequences, infiltration of leukemic cells into the bone marrow can also manifest abnormalities in diffusion-weighted images (DWI) and apparent diffusion coefficient (ADC) [16]. Differential considerations between inflammation and leukemia may also be supported by distribution of radiological findings. While focal alterations mainly occur in patients with JIA, osteomyelitis or lymphoma, diffuse bone marrow abnormalities on MRI are usual in patients with leukemia [17]. Reported predilection sites include the pelvic girdle and the thoracolumbar spine [18].

Despite the growing use of MRI in pediatric musculoskeletal diagnostics, systematic descriptions of MRI findings preceding the diagnosis of ALL remain limited. The following observational study aims to highlight the role of MRI in the early detection of childhood leukemia, especially in patients where blood counts are non-diagnostic. Additionally, we aim to define typical alterations on MRI in children with newly diagnosed ALL that justify timely bone marrow examination. We therefore report a retrospective single-center analysis of children with newly diagnosed ALL who underwent MRI before diagnostic confirmation of ALL.

## Methods

### Study design and participants

We conducted a retrospective, single-center analysis of pediatric patients (0–18 years) diagnosed with ALL between 2006 and 2024. We included all newly diagnosed ALL patients who received an MRI as part of the initial diagnostic work-up.

### Data collection

Clinical data, including age, gender, underlying conditions, and initial presenting symptoms, were collected from medical records. Laboratory data included hemoglobin, white blood cell (WBC) count, platelet count, C-reactive protein (CRP; mg/dL), Lactate dehydrogenase (LDH, U/L), peripheral blood blasts (%), and bone-marrow blasts (%). All MRI studies were reviewed by a pediatric radiologist with more than 30 years of clinical experience in interpreting pediatric MRI images. Leukemia subtypes by flow cytometry as well as the presence of typical genetic alterations found in childhood ALL were recorded when available. Flow cytometry of peripheral blood and bone marrow was performed at our institution using pre-formulated dry-reagent tubes from two product lines: DURAClone RE ALB (B-ALL panel) and the ClearLLab T-cell tube (ClearLLab panel family), following manufacturer protocols [19, 20]. Outcome of leukemia treatment was documented by treatment stratification, minimal residual disease (MRD) during treatment and occurrence of relapse.

### Data analysis

Descriptive statistics were used to summarize patient demographics, clinical characteristics, and MRI findings. We summarized laboratory values using mean values. Diagnostic intervals were calculated in days (symptom onset until MRI; MRI until bone marrow puncture) and reported as mean. MRI scans were reviewed for predefined bone marrow signal alterations, including T1w hypointensity, PDw or T2w-STIR hyperintensity, high DWI signal with low ADC, and distribution patterns (diffuse vs. multifocal/focal).

## Results

Cohort: 20 patients were included in the study, with a male-to-female ratio of 12:8 and a median age at diagnosis of 7.8 years (range 1–17 years). Of 424 newly diagnosed ALL cases at our center (2006–2024), 20 (4.7%) were prompted by suspicious MRI findings. 8 of 20 patients were studied at our center, in 12 patients the MRI examinations were performed in 5 different referring hospitals and 2 radiology practices before admission in our clinic. In total 24 MRI examinations were available in 20 patients. 21 Examinations focused on the musculoskeletal system in 17 patients. The remaining examinations consisted of three MRI examinations of chest and/or abdomen.

All examinations followed standard imaging protocols. A key difference in musculoskeletal imaging was that each protocol consistently included a standard T1-weighted turbo spin-echo (T1w-TSE) sequence, complemented by either a proton-density-weighted (PDw) sequence or a T2-weighted short tau inversion recovery (T2w-STIR) sequence. In contrast, in chest and abdominal MRI examinations, these sequences were only applied in one out of three cases. Conversely, diffusion-weighted imaging (DWI) was performed in all chest and abdominal studies, whereas in musculoskeletal imaging it was used in only three out of seventeen cases.

### Clinical presentation and radiological findings

**Fig. 1:**
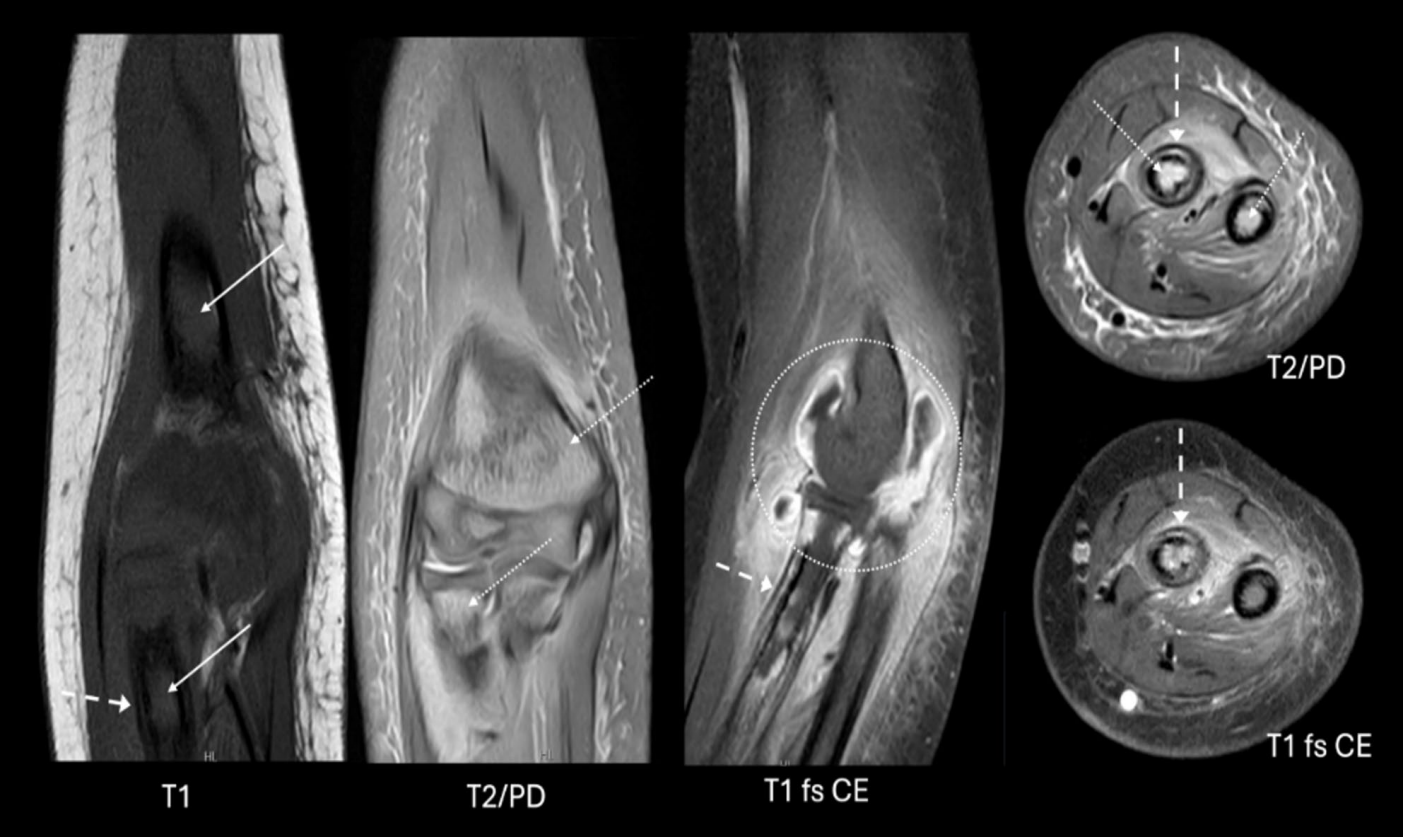
Patient 12. MRI of the right elbow shows bone marrow signal alterations characterized by T1w hypointensity (arrows) and T2w hyperintensity (dotted arrows). Joint effusion (dotted circle) and periosteal reaction (dashed arrow) are also indicated. This case is representative of the diagnostic challenge because inflammatory findings initially suggested osteomyelitis, whereas the diffuse marrow abnormalities ultimately prompted hematologic evaluation

In our cohort, the mean interval from symptom onset to MRI referral was 20.8 days (range 1–65 days, see Fig. 4A). The main symptom preceding imaging was bone pain in 18/20 patients, without specific pain localization; pain occurred in all extremities, hips, and spine. MRI was predominantly prescribed to evaluate suspected osteomyelitis, mostly in the presence of normal blood counts. Patient P12, a 4-year-old boy with Down syndrome, illustrates this typical presentation. He presented with pain and swelling of the right elbow joint and was initially diagnosed with osteomyelitis based on arthritis and synovitis findings on MRI (Fig. 1). Antibiotic treatment was initiated with clindamycin and cefuroxime. Around one week later, after no clinical benefit, re-evaluation of the MRI by specialized pediatric radiologists prompted bone marrow puncture because of extensive T1w hypointensity and T2w hyperintensity of the bone marrow. Notably, P12 had undergone bone marrow puncture and biopsy four months before diagnosis because of transient bicytopenia (leukocytes: 3.2 × 10⁹/L; platelets: 50 × 10⁹/L). Both aspirate and biopsy showed no leukemic blasts at that time. Due to mild signs of dysplasia and hypocellularity, a myelodysplastic syndrome was suspected. None of the patients in our cohort were referred for MRI because leukemia or another hematological malignancy was suspected.

All patients underwent a CBC prior to imaging. A completely normal CBC was present in 7/20 patients (35%), and 13/20 had a normal white blood cell count. In a further 6/20 patients, mild anemia was the only abnormality. The mean hemoglobin level was 10.1 g/dL (range 3.4–13 g/dL), the mean leukocyte count was 8.54 × 10⁹/L (range 1.2–33 × 10⁹/L), and the mean platelet count was 270 × 10⁹/L (range 31–812 × 10⁹/L). Mean C-reactive protein and LDH levels were moderately elevated at 6.14 mg/dL (range < 0.1–24.7 mg/dL) and 462 U/L (range 171–2280 U/L), respectively. An overview of all laboratory findings is provided in Table 1.

**Table 1:** Laboratory findings and incidence of osteonecrosis

Patient	WBC (x10^9^/L)	Hemoglobin (g/dL)	Platelet (x10^9^/L)	CRP (mg/dl)	LDH(U/l)	Osteonecrosis
P1	7.5	12.0	167	3.0	329	No
P2	6.2	12.9	300	1.3	380	No
P3	4.6	7.7	812	8.0	254	No
P4	8.7	12.3	200	0	277	Yes
P5	5.8	13.0	279	1.0	224	No
P6	9.2	12.8	347	13.0	627	No
P7	8.6	10.0	327	15.0	308	No
P8	3.8	9.9	93	0.6	432	No
P9	6.5	8.7	238	24.7	721	No
P10	3.8	10.9	118	6.3	256	No
P11	6.8	11	408	8.0	171	Yes
P12	5.1	11.7	261	16.5	306	Yes
P13	20.3	8.7	63	5.1	607	No
P14	33.3	11.8	259	12.1	2280	No
P15	5.0	4.3	31	0.6	252	No
P16	2.3	11.8	76	5.9	203	No
P17	1.2	11.2	190	1.0	188	No
P18	18.9	8.2	267	0	690	No
P19	8.4	3.4	333	< 0.1	293	No
P20	4.8	9.9	626	0.5	450	No

*WBC* white blood cell, *PB* peripheral blasts, *CRP* C-reactive protein level, *LDH* Lactate dehydrogenase

**Fig. 2:**
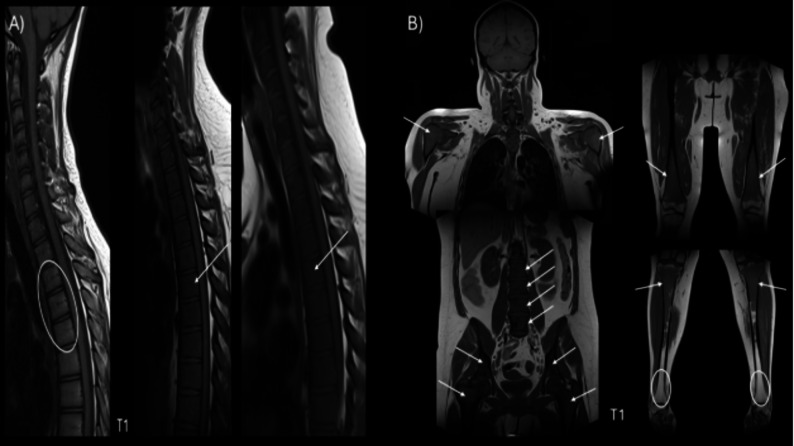
**(A)** Sagittal spinal MR images in patient 2 show diffuse T1w hypointensity in the spinal bone marrow (arrows) at first presentation (middle image) and six months later (right image), compared with normal marrow signal in a healthy control (circle, left image). **(B)** Whole-body MRI in patient 2, performed six months after the initial presentation, demonstrates diffuse T1w hypointensity in the bone marrow of the pelvis, spine, and upper and lower extremities (arrows), while normal marrow signal is preserved in the distal tibiae. This case illustrates that characteristic MRI abnormalities may precede diagnostic peripheral blood or bone marrow findings and may otherwise be followed by a prolonged diagnostic delay

The interval between MRI and subsequent hematologic evaluation varied widely (mean 18.4 days; range 0–180 days, see Fig. 4B). Peripheral blood blasts were present in 8/20 cases, with a mean of 6.2% (range 0–30%), while bone marrow blasts ranged from 0% to 91%, with a mean of 59.4% (in one patient, ALL was diagnosed only via bone biopsy, which demonstrated 75% blasts). Highlighting the diagnostic challenge when peripheral blood remains non-diagnostic (leukocytes 6.2 × 10⁹/L, hemoglobin 12.9 g/dL, platelets 300 × 10⁹/L), patient P2, a 17-year-old girl with persistent back pain, underwent MRI to evaluate suspected disc pathology. MRI showed diffuse marrow signal abnormalities with T1w hypointensity and T2w-STIR hyperintensity (Fig. 2A). Initial peripheral blood work-up showed no sign of leukemia, leading to corticosteroid therapy for presumed rheumatologic disease. Six months later, due to persistent pain, bone marrow aspiration and biopsy were conducted. After negative flow cytometry, a second MRI scan was obtained (Fig. 2B). B-ALL was eventually established by bone marrow biopsy only days after the second scan.

**Fig. 3:**
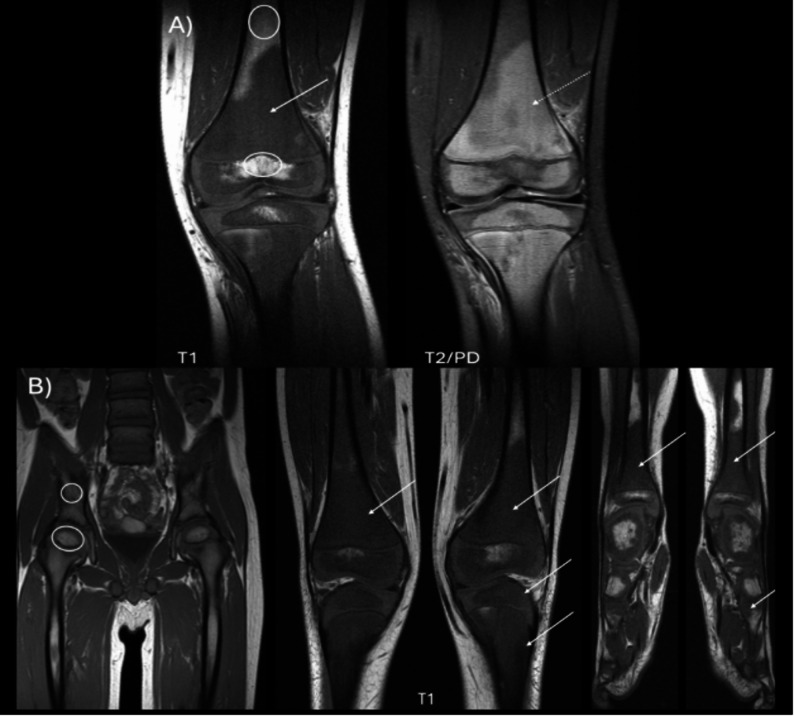
**(A)** In patient 5, MRI of the left knee shows pathologically low T1w signal (arrow) and high T2w signal intensity in the bone marrow (dotted arrow), compared with normal marrow (circles). **(B)** In the same patient, MR images from the second examination, performed eight days later, show normal pelvic bone marrow (circles), whereas the bone marrow of both knees and distal lower legs demonstrates confluent lesions with complete loss of fatty marrow (arrows). This case is representative of the diagnostic challenge because an initial iliac bone marrow puncture showed no evidence of leukemia; MRI-guided biopsy from the left tibia subsequently confirmed ALL

To illustrate the potential utility of MRI in guiding bone marrow biopsy site selection, P5, a 5-year-old boy presenting with atraumatic knee swelling and initially unremarkable blood counts, underwent MRI for suspected osteomyelitis. Imaging revealed diffuse marrow signal abnormalities (T1w hypointensity and T2w hyperintensity; Fig. 3A). Initial iliac bone marrow aspiration yielded no blasts; however, repeat MRI eight days later demonstrated persistent and progressive signal alterations (Fig. 3B), prompting tibial re-biopsy, which confirmed B-ALL with 55% blast infiltration.

Bone marrow signal changes (summarized in Table 2) were present in all 17 patients who underwent musculoskeletal examinations. The most common findings were T1w signal loss in all cases and PDw or T2w-STIR hyperintensity in 16/17 cases. In the three patients examined with DWI and ADC, each demonstrated diffusion restriction with low ADC values in multiple bones. Standard T2-weighted turbo spin-echo sequences did not contribute to identification of leukemia. The diagnostic value of additional contrast-enhanced T1w sequences in nine cases was very low; no examination identified additional findings relevant to diagnosis or treatment.

**Table 2:** MRI findings combined with MRD, genetics and bone marrow infiltration

Patient	% blasts in BM	Main MRI Signal alterations					Genetics	MRD at TP1
		T1w	PDw/T2w-STIR	DWI/ADC	diffuse/confluent	focal/multifocal		
P1	70	↓	↑	↑ / ↓	X	-	High hyperdiploidy	< 10^− 4^
P2	0	↓	↑	nd	X	-	-	< 10^− 4^
P3	40	↓	↑	nd	X	-	-	10^− 4^
P4	89	↓	↑	nd	X	X in parts	-	10^− 2^
P5	55	↓	↑	nd	X	-	-	10^− 4^
P6	53	↓	↑	↑ / ↓	X	X in parts	Hypodiploidy	Positive, not quantified
P7	69	↓	↑	nd	X	-	-	Positive, not quantified
P8	91	↓	↑	nd	X	-	*ETV6*::*RUNX1*	7 × 10^− 4^
P9	33	↓	↑	nd	X	-	-	Positive, not quantified
P10	76	↓	↑	nd	X	-	*ETV6*::*RUNX1*	Positive, not quantified
P11	70	↓	↑	nd	X	-	*ZNF384::EP300*	10^− 3^
P12	75.4	↓	↑	nd	X	-	*TCF3*::*PBX1*	Negative
P13	80	↓	↑	nd	X	-	*CRLF2*::*P2RY8*	-
P14	56.7	↓	↑	↑ / ↓	X	X in parts	*TCF3*::*PBX1*	< 10^− 5^
P15	82.5	↓	↑	nd	X	-	-	Negative
P16	70	↓	↑	nd	X	-	-	Negative
P17	80	↓	↑	nd	X	-	-	Negative
P18	17	nd	nd	↑ / ↓	X	Kidneys +Testes	*PAX5*::*SOX5*	Negative
P19	51	↓	nd	↑ / ↓	X	Kidneys	-	Negative
P20	30	nd	nd	↑ / ↓	X	Peritoneal cavity	-	Negative

*BM* bone marrow, *PDw* proton-density-weighted, *T2w-STIR* T2-weighted short tau inversion recovery, *DWI* diffusion-weighted imaging, *ADC* apparent diffusion coefficient, *MRD* minimal residual disease, *TP1* time point 1; nd: not done

All patients showed diffuse, occasionally widespread bone marrow signal abnormalities, often with confluent metaphyseal lesions. Three patients exhibited additional multifocal bone marrow lesions. In the children with examinations of the trunk, the most common findings were diffusion restriction of bone marrow and/or other organs or compartments (kidneys, testis, intraperitoneal tumor mass) with low ADC values (3 of 3). In abdominal imaging, T2w sequences (HASTE, TrueFISP) demonstrated multifocal tumors of kidneys or in the peritoneal cavity (3 of 3).

### Disease characteristics and outcome

Most patients did not have preceding medical conditions. Four patients had concomitant genetic diseases: two patients were already diagnosed with Trisomy 21, one patient underwent genetic testing after the diagnosis of leukemia and was found to have a Li-Fraumeni syndrome, and the fourth was already under hematological outpatient care because of a spherocytosis. All patients showed a B-cell precursor ALL. Notably, there was no case of T-cell ALL (T-ALL) or acute myeloid leukemia (AML) presenting with MRI-diagnosed bone marrow abnormalities. Only one patient showed involvement of the central nervous system. Additionally, there was no association with a specific genetic subtype. Standardized genetic testing revealed *ETV6*::*RUNX1* fusion in three patients, hyperdiploidy of chromosomes in two patients and hypodiploidy in one patient. In addition, the following gene fusions were identified: *TCF3*::*PBX1* (*n* = 2), *CRLF2*::*P2RY8* (*n* = 1), *PAX5*::*SOX5* (*n* = 1), and *ZNF384*::*EP300* (*n* = 1). None of the patients had *MLL* or *BCR*::*ABL* rearrangements.

Patients were treated according to AIEOP-BFM (2000, 2009 and 2017) and CoALL study protocols. Three patients were assigned to high-risk treatment arms due to measurable minimal residual disease (MRD) after consolidation. All 20 patients survived cancer treatment. Three patients experienced relapse, and two patients developed secondary malignancies (T-ALL and lymphoma). Again, there was no association with a specific risk group, and the presence of radiological bone marrow abnormalities was not a predictive marker for further treatment response. Furthermore, three patients developed osteonecrosis during the treatment period.

## Discussion

In this single center ALL cohort of 424 patients, suspicion of the underlying ALL was prompted by MRI in 20 patients. More than one-third of this diagnostically challenging subgroup (7/20; 35%) had a completely normal CBC at presentation, and peripheral blood assessment did not detect blasts in most children (11/20). The initial radiological suspicion was driven primarily by T1w bone marrow hypointensity. In most cases, normal or non-conclusive blood counts delayed recognition, whereas bone marrow examination was conducted soon after MRI in most patients and confirmed the radiological suspicion. Thus, a normal CBC cannot reliably exclude ALL in a child with persistent musculoskeletal symptoms and a suspicious marrow pattern on MRI.

**Fig. 4:**
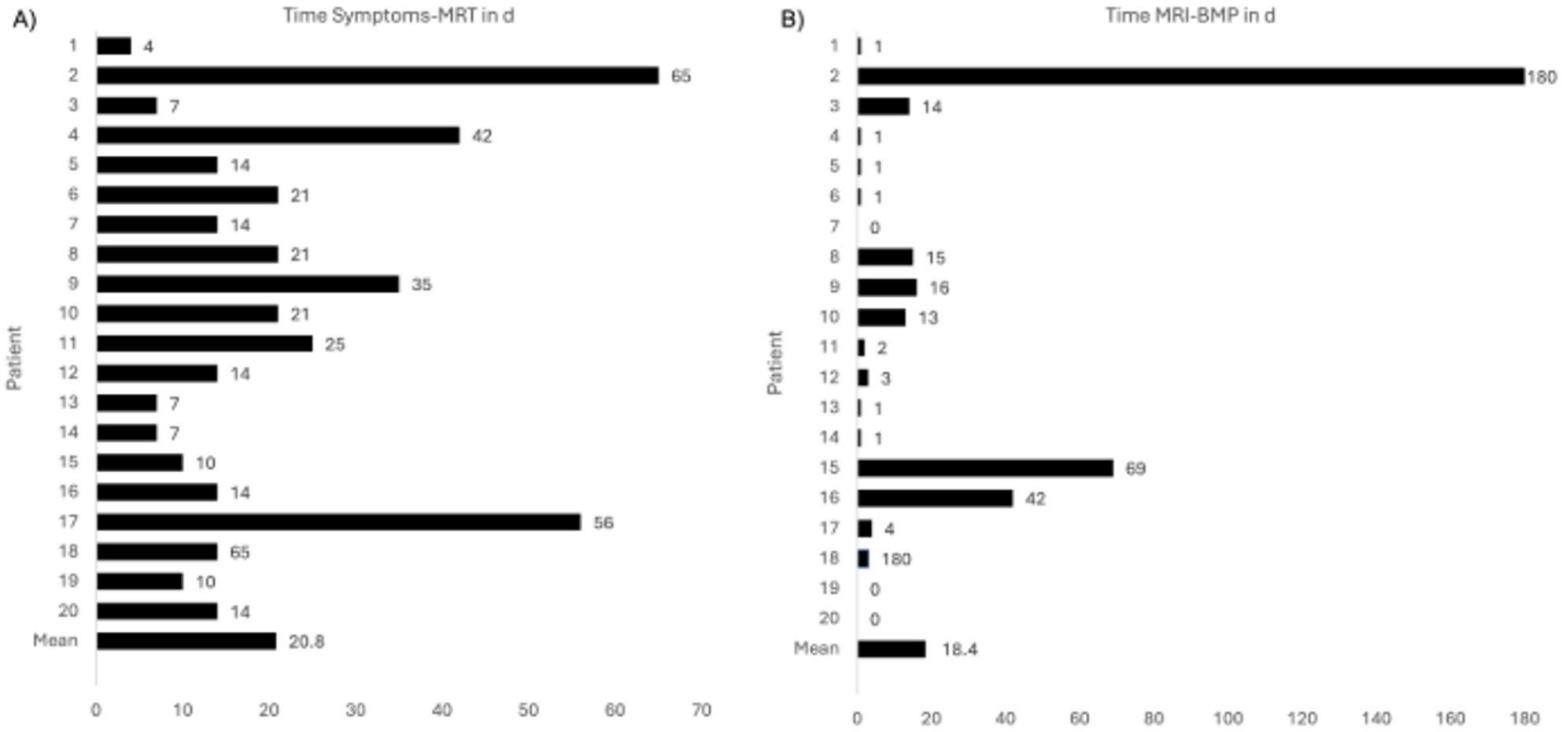
Horizontal bar charts showing diagnostic time intervals (days) for each patient in the cohort (*n* = 20). **(A)** Time from onset of symptoms to MRI. **(B)** Time from MRI to bone marrow puncture (BMP)

In our analysis, the time between onset of symptoms and MRI was 20.8 days (Fig. 4A), highlighting the difficulty of ALL diagnosis in children with leading MSK symptoms and absence of clear blood count abnormalities. Diagnostic delay extended up to 65 days with several patients experiencing delays exceeding five weeks before undergoing MRI. These prolonged delays were largely attributable to initially mild, fluctuating symptoms, or misattribution to orthopedic, developmental, or inflammatory conditions. The prolonged delay in diagnosis of ALL in patients with only MSK symptoms was also recently shown by Lyngdoh et al., who published a large retrospective study showing that children mainly presenting with MSK symptoms exhibited delayed diagnosis of leukemia (4 versus 2 weeks) [21]. Whereas Lyngdoh et al. emphasized laboratory criteria to distinguish ALL from other differentials, our findings highlight the complementary and often decisive role of MR imaging when laboratory parameters are inconclusive.

Importantly, in our cohort, the interval from MRI to hematologic work-up varied widely, ranging from 0 to 180 days (Fig. 4B). This variability was predominantly driven by radiologic misinterpretation, with several MRI scans initially read as osteomyelitis or even normal. Consequently, some patients received antibiotic therapy prior to further diagnostic work-up, delaying leukemia diagnosis. Either worsening of MSK symptoms or the emergence of blood count abnormalities during therapy prompted further diagnostic work-up or reevaluation of MRI findings. Finding more objective and quantitative measurements for the presence of leukemic infiltration of the bone marrow could lead to less misinterpretation. Zadig et al. studied the intra- and interobserver reliability of a scoring system for assessment of high signal areas within the bone marrow on T2-weighted images [22]. They showed that grading the intensity and extent of hyperintensity was reliable within and between different observers. Beyond qualitative scoring systems, quantitative measurements, such as the apparent diffusion coefficient (ADC) derived from diffusion-weighted imaging, may further reduce misinterpretation. Ibrahim et al. demonstrated that ADC values are significantly lower in leukemic bone marrow infiltration compared to normal red marrow, with an optimal cutoff of 0.612 × 10⁻³ mm²/s for differentiating malignant from benign marrow signal in pediatric patients with hematologic malignancies [23]. Building on these findings, adopting standardized descriptors and a harmonized scoring system for bone-marrow signal intensity could enhance consistency when evaluating children with musculoskeletal symptoms.

The most frequent bone marrow signal alteration in our cohort was diffuse T1w hypointensity, followed by PDw or T2w-STIR hyperintensity. These changes are most likely due to reduced marrow fat resulting from leukemic infiltration [24]. Distribution was diffuse in 14/17 patients with musculoskeletal MRI, consistent with systemic marrow involvement. Our findings are in line with prior pediatric case series reporting early marrow signal alterations on MRI in leukemia. Kato et al. demonstrated that MRI-detectable marrow changes may precede diagnostic bone marrow aspiration [25]. Our cohort extends these observations, including one patient in whom MRI abnormalities consistent with leukemia were present up to six months before diagnostic confirmation, providing further evidence that radiological marrow changes may emerge early in disease evolution.

In addition to alterations on conventional sequences, diffusion-weighted imaging revealed further abnormalities: High DWI signal with low ADC was observed in 6/6 patients. These findings are consistent with previous reports by Nishii et al. and Cao et al., who described characteristic diffusion abnormalities in the skull of pediatric patients with leukemia. Notably, these studies also demonstrated normalization of diffusion parameters during treatment, suggesting that MRI may have utility not only in the initial diagnosis but also in monitoring disease response [16, 26].

When comparing clinical features, no increased risk of osteonecrosis (a common side effect of cortisone treatment) was shown in our cohort despite initial, symptomatic involvement of the skeletal system [27]. Analysis of cytogenetic subgroups revealed distributions largely consistent with established pediatric ALL benchmarks (*ETV6*::*RUNX1* 18% vs. 20–25%, hyperdiploidy 12% vs. 25–30%, *TCF3*::*PBX1* 12% vs. 3–5%, *KMT2A* 0% vs. 3–5%, *BCR::ABL1* 0% vs. 2–3%) [28]. The slightly higher frequency of *TCF3*::*PBX1* and lower frequency of hyperdiploidy are likely due to the small sample size rather than a true enrichment; within our data, genotype did not seem to correlate to a MSK manifestation or a distinct MRI pattern.

**Fig. 5:**
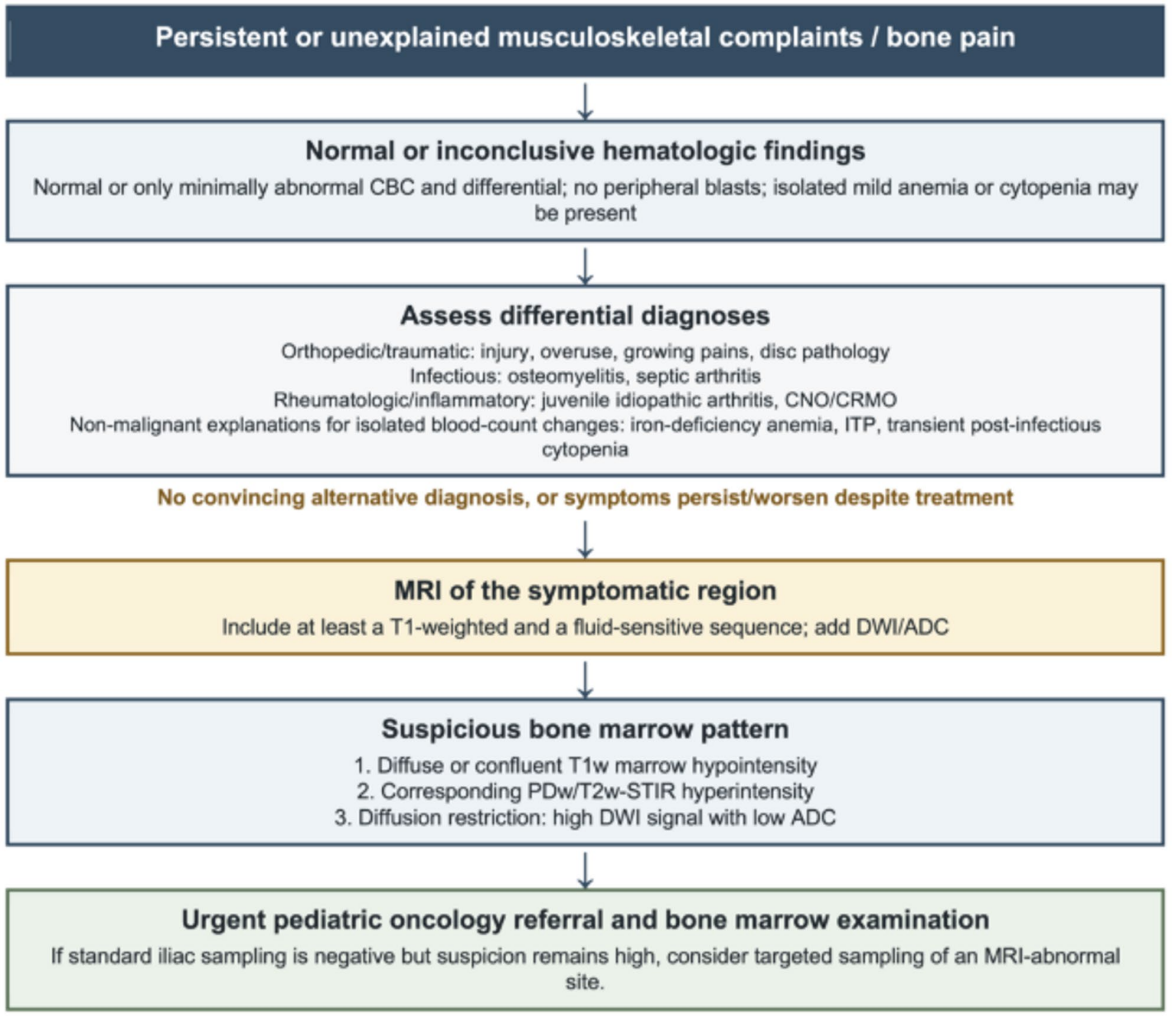
Proposed clinical pathway for children and adolescents with persistent musculoskeletal complaints and normal or inconclusive initial hematologic findings. This pathway is based on the present retrospective cohort and requires prospective validation. ADC, apparent diffusion coefficient; CBC, complete blood count; CNO/CRMO, chronic nonbacterial osteomyelitis/chronic recurrent multifocal osteomyelitis; DWI, diffusion-weighted imaging; ITP, immune thrombocytopenia; MRI, magnetic resonance imaging; MSK, musculoskeletal; PDw, proton-density-weighted; T1w, T1-weighted; T2w-STIR, T2-weighted short tau inversion recovery

Regarding clinical management, diffuse or confluent T1w marrow hypointensity, particularly when accompanied by PDw or T2w-STIR hyperintensity, diffusion restriction, multifocal lesions, or findings discordant with a presumed focal inflammatory process, should be treated as a red flag in a child with persistent or unexplained musculoskeletal pain. These findings should prompt urgent expert radiological review and referral to pediatric oncology, even when the CBC is normal or only minimally abnormal. Bone marrow aspiration should then be considered by the oncology team. Targeted sampling of an MRI-abnormal site, especially when standard iliac sampling is negative but clinical and radiological suspicion remains high, may be useful, as illustrated by patient P5. A proposed clinical pathway is summarized in Fig. 5.

This study has several limitations. Its retrospective single-center design, the small sample of 20 patients, and heterogeneous MRI protocols across the referring institutions limit generalizability. Because we included only patients with ALL who underwent MRI before diagnostic confirmation and did not include a control group, the study cannot determine how reliably these findings distinguish ALL from benign or inflammatory marrow alterations. Nevertheless, these patients represent a rare but clinically important subgroup in which routine laboratory findings may be falsely reassuring.

Future prospective multicenter studies should validate these MRI patterns and the proposed clinical pathway in larger cohorts that include relevant inflammatory and orthopedic differential diagnoses. Whole-body MRI may help characterize the distribution of marrow involvement and guide biopsy site selection, while quantitative MRI parameters, particularly ADC values, may not only reduce observer-dependent interpretation, but might also help distinguish between different differential diagnoses. Prospective studies should determine age-appropriate quantitative thresholds and assess whether these approaches shorten the time to diagnosis.

## Conclusions

MRI may provide the first indication of pediatric ALL in children presenting with persistent musculoskeletal symptoms and non-diagnostic blood counts. Recognizing diffuse T1w marrow hypointensity, with or without diffusion restriction, is crucial. In our cohort, 35% of patients had a completely normal CBC at presentation; therefore, a normal CBC should not delay urgent pediatric oncology referral and consideration of bone marrow examination when the MRI pattern is suspicious.

## Data Availability

The dataset used and analyzed during the current study are available from the corresponding author on reasonable request.

## Abbreviations

MRI: Magnetic resonance imaging
ALL: Acute lymphoblastic leukemia
CBC: Complete blood count
JIA: Juvenile idiopathic arthritis
CRP: C-reactive protein
MSK: Musculoskeletal
ADC: Apparent diffusion coefficient
DWI: Diffusion-weighted imaging
WBC: White blood cell
MRD: Minimal residual disease
LDH: Lactate dehydrogenase
FLAIR: Fluid-attenuated inversion recovery
PDw: Proton-density-weighted
T1w: T1-weighted
T2w-STIR: T2-weighted short tau inversion recovery
AML: Acute myeloid leukemia
BMP: Bone marrow puncture

## References

[1] Deutsches Kinderkrebsregister (2023) Jahresbericht 2022. Institut für Medizinische Biometrie, Epidemiologie und Informatik, Mainz

[2] Moussavi F, Hosseini SN, Saket S, Derakhshanfar H (2014) The first CBC in diagnosis of childhood acute lymphoblastic leukemia. J Emerg Health Care 3(1):9–12

[3] Meehan PL, Viroslav S, Schmitt EW Jr (1995) Vertebral collapse in childhood leukemia. J Pediatr Orthop 15(5):592–595 7593568 10.1097/01241398-199509000-00008

[4] Jonsson OG, Sartain P, Ducore JM, Buchanan GR (1990) Bone pain as an initial symptom of childhood acute lymphoblastic leukemia: association with nearly normal hematologic indexes. J Pediatr 117(2):233–237 2380822 10.1016/s0022-3476(05)80535-9

[5] Mangum DS, Bishop JD, Zhou Y, Cheng C, Karol SE, Rubnitz JE et al (2023) Characterisation of children and adolescents with acute lymphoblastic leukaemia who presented without peripheral blood blasts at diagnosis. Br J Haematol 200(3):338–343 36352514 10.1111/bjh.18552 PMC9852218

[6] Clarke RT, Van den Bruel A, Bankhead C, Mitchell CD, Phillips B, Thompson MJ (2016) Clinical presentation of childhood leukaemia: a systematic review and meta-analysis. Arch Dis Child 101(10):894–901. 10.1136/archdischild-2016-311251 27647842 10.1136/archdischild-2016-311251

[7] Giraudo C, Fichera G, Michielin A, Zulian F, Stramare R, Rennie WJ (2024) Bone marrow edema in children: chronic nonbacterial osteomyelitis and its mimickers. Ther Adv Musculoskelet Dis 16:1759720X241278438. 10.1177/1759720X241278438 39314820 10.1177/1759720X241278438 PMC11418244

[8] Tsujioka T, Sugiyama M, Ueki M, Tozawa Y, Takezaki S, Ohshima J et al (2018) Difficulty in the diagnosis of bone and joint pain associated with pediatric acute leukemia; comparison with juvenile idiopathic arthritis. Mod Rheumatol 28(1):108–113. 10.1080/14397595.2017.1332474 28612674 10.1080/14397595.2017.1332474

[9] Louvigné M, Rakotonjanahary J, Goumy L, Tavenard A, Brasme JF, Rialland F et al (2020) Persistent osteoarticular pain in children: early clinical and laboratory findings suggestive of acute lymphoblastic leukemia (a multicenter case-control study of 147 patients). Pediatr Rheumatol Online J 18(1):1. 10.1186/s12969-019-0376-8 31898528 10.1186/s12969-019-0376-8 PMC6941319

[10] Brix N, Glerup M, Foell D, Kessel C, Wittkowski H, Berntson L et al (2023) Inflammatory biomarkers can differentiate acute lymphoblastic leukemia with arthropathy from juvenile idiopathic arthritis better than standard blood tests. J Pediatr 258:113406. 10.1016/j.jpeds.2023.113406 37023943 10.1016/j.jpeds.2023.113406

[11] Organisation for Economic Co-operation and Development (OECD) (2023) Diagnostic technologies. Health at a Glance 2023: OECD Indicators. OECD Publishing, Paris, pp 116–117. doi:10.1787/7a7afb35-en.

[12] Tannenbaum MF, Noda S, Cohen SA, Rissmiller JG, Golja AM, Schwartz DM (2020) Imaging musculoskeletal manifestations of pediatric hematologic malignancies. AJR Am J Roentgenol 214(2):455–464. 10.2214/AJR.19.21833 31799868 10.2214/AJR.19.21833

[13] Guillerman RP (2008) Imaging of normal and abnormal bone marrow. In: Slovis TL, Adler BH, Bloom DA et al (eds) Caffey’s Pediatric Diagnostic Imaging, 11th edn. Elsevier, Philadelphia, pp 2970–2996

[14] Person A, Janitz E, Thapa M (2021) Pediatric bone marrow: normal and abnormal MRI appearance. Semin Roentgenol 56(3):325–337. 10.1053/j.ro.2021.05.002 34281683 10.1053/j.ro.2021.05.002

[15] Yoshikawa T, Tanizawa A, Suzuki K, Tanaka N, Hayashi T, Tsuda M et al (2016) Usefulness of T1-weighted magnetic resonance images for diagnosis of acute leukemia manifesting musculoskeletal symptoms prior to appearance of peripheral blood abnormalities. Case Rep Pediatr 2016:2802596. 10.1155/2016/2802596 27830102 10.1155/2016/2802596 PMC5088267

[16] Nishii T, Kono AK, Akasaka Y, Mori T, Hayakawa A, Iijima K et al (2015) Bone marrow magnetic resonance imaging of the clivus in pediatric leukemia patients and normal controls. Jpn J Radiol 33(3):146–152. 10.1007/s11604-015-0394-5 25652242 10.1007/s11604-015-0394-5

[17] Nguyen JC, Davis KW, Arkader A, Guariento A, Sze A, Hong S et al (2021) Pre-treatment MRI of leukaemia and lymphoma in children: are there differences in marrow replacement patterns on T1-weighted images? Eur Radiol 31(10):7992–8000. 10.1007/s00330-021-07814-z 33768286 10.1007/s00330-021-07814-z

[18] Silva JR Jr, Hayashi D, Yonenaga T, Fukuda K, Genant HK, Lin C et al (2013) MRI of bone marrow abnormalities in hematological malignancies. Diagn Interv Radiol 19(5):393–399. 10.5152/dir.2013.067 23748035 10.5152/dir.2013.067

[19] Hedley BD, Cheng G, Keeney M, Kern W, Padurean A, Luider J et al (2021) A multicenter study evaluation of the ClearLLab 10 C panels. Cytometry B Clin Cytom 100(2):225–234. 10.1002/cyto.b.21935 32667744 10.1002/cyto.b.21935 PMC8048967

[20] Swart L, Pretorius M, Lawrie D, Glencross D (2022) Commercial DURAClone panels for extending the repertoire of multicolour immunophenotypic panels in an academic flow cytometry laboratory in South Africa. Afr J Lab Med 11(1):e1720. 10.4102/ajlm.v11i1.1720 10.4102/ajlm.v11i1.1720 PMC9724122 36483322

[21] Lyngdoh KS, Dhruti P, Totadri S et al (2025) The implications of musculoskeletal manifestations in acute lymphoblastic leukemia: a decade’s experience from a referral center in southern India. Indian Pediatr 62:489–494. 10.1007/s13312-025-00064-y 40193032 10.1007/s13312-025-00064-y

[22] Zadig P, von Brandis E, d’Angelo P, de Horatio LT, Ording-Müller LS, Rosendahl K et al (2022) Whole-body MRI in children aged 6–18 years: reliability of identifying and grading high signal intensity changes within bone marrow. Pediatr Radiol 52(7):1272–1282. 10.1007/s00247-022-05312-y 35445816 10.1007/s00247-022-05312-y PMC9192437

[23] Ibrahim YA, Elsadawy ME, El Naggar T (2019) Role of quantitative diffusion-weighted imaging in differentiation between red and infiltrated marrow in pediatric patients with hematologic malignancy. Egypt J Radiol Nucl Med 50:13. 10.1186/s43055-019-0017-8

[24] Luitjens J, Baur-Melnyk A (2021) Skelettveränderungen bei hämatologischen Systemerkrankungen. Radiologe 61:1068–1077. 10.1007/s00117-021-00934-z 34820696 10.1007/s00117-021-00934-z

[25] Kato M, Koh K, Kikuchi A, Toyama D, Mochizuki S, Uchisaka N et al (2011) Case series of pediatric acute leukemia without a peripheral blood abnormality, detected by magnetic resonance imaging. Int J Hematol 93(6):787–790. 10.1007/s12185-011-0842-7 21509438 10.1007/s12185-011-0842-7

[26] Cao W, Liang C, Gen Y, Wang C, Zhao C, Sun L (2016) Role of diffusion-weighted imaging for detecting bone marrow infiltration in skull in children with acute lymphoblastic leukemia. Diagn Interv Radiol 22(6):580–586. 10.5152/dir.2016.15167 27763327 10.5152/dir.2016.15167 PMC5098955

[27] Brivio E, Cossio A, Borra D, Silvestri D, Prunotto G, Colombini A et al (2022) Osteonecrosis in paediatric acute lymphoblastic leukaemia: incidence, risk factors, radiological patterns and evolution in a single-centre cohort. Br J Haematol 197(5):602–608. 10.1111/bjh.18147 35362095 10.1111/bjh.18147 PMC9323502

[28] Inaba H, Mullighan CG (2020) Pediatric acute lymphoblastic leukemia. Haematologica 105(11):2524–2539. 10.3324/haematol.2020.247031 33054110 10.3324/haematol.2020.247031 PMC7604619

